# GGA2 interacts with EGFR cytoplasmic domain to stabilize the receptor expression and promote cell growth

**DOI:** 10.1038/s41598-018-19542-4

**Published:** 2018-01-22

**Authors:** Takefumi Uemura, Satoshi Kametaka, Satoshi Waguri

**Affiliations:** 1 Department of Anatomy and Histology, Fukushima Medical University School of Medicine, 1 Hikariga-oka, Fukushima City, Fukushima, 960-1295 Japan; 2 Department of Physical Therapy, Nagoya University Graduate School of Medicine, 1-1-20 Daiko-minami, Higashi, Nagoya City, Aichi 461-8673 Japan

## Abstract

Epidermal growth factor receptor (EGFR) signaling and its downregulation upon ligand binding have been extensively documented. However, the mechanisms by which cells maintain steady-state EGFR expression remain poorly understood. Here, we report a novel role of Golgi-localized, γ-adaptin ear-containing, ADP ribosylation factor-binding protein 2 (GGA2) in the control of EGFR turnover. Whereas GGA1- or GGA3-depletion increased EGFR expression, GGA2-depletion by RNAi greatly reduced steady-state expression of EGFR, reflecting enhanced lysosomal degradation of EGFR. Subsequent pull-down assays showed interactions of VHS-GAT domains from three GGAs with the cytoplasmic juxtamembrane region (jxt) of EGFR, which was dependent on N108 in the VHS domain. Proximity ligation assay also revealed the steady-state interaction between GGA2 and EGFR *in situ*. Moreover, reduced expression of EGFR in GGA2-depleted cells was reversed by additional depletion of GGA1 or GGA3, suggesting that GGA1 and GGA3 promote EGFR degradation. In addition, GGA2-depleted cells had reduced EGF signaling and cell proliferation in cell culture and xenograft experiments. Finally, GGA2 was upregulated in 30.8% of human hepatocellular carcinomas and 23.3% of colorectal cancers. Together, these results indicate that GGA2 supports cell growth by interacting with EGFR for sustaining the receptor expression.

## Introduction

Epidermal growth factor receptor (EGFR; also known as ErbB1 or Her1) is a receptor tyrosine kinase (RTK) that is involved in eukaryotic development and cancer pathogenesis^1^. Several sequential and parallel events reportedly occur upon EGF binding, including EGFR dimerization and activation of signal cascades, endocytosis, ubiquitination, recycling back to plasma membrane, sorting into inner vesicles of multivesicular bodies (MVBs), and lysosomal degradation, and these processes of maintenance and suppression of EGFR have been documented under EGF signaling conditions^2,3^. However, the regulatory mechanisms of steady-state EGFR expression in the absence of EGF stimulation remain poorly understood. Although this concept has been overlooked, it has become evident that gene amplification, overexpression, mutation, and aberrant activation of EGFR are frequently found in many human cancers^4^.

The GGA proteins GGA1, GGA2, and GGA3 are a family of ubiquitously expressed ADP ribosylation factor (ARF)-dependent monomeric clathrin adaptors that are conserved from yeast to humans. All GGA proteins comprise three folded domains of VHS, GAT, and GAE and a hinge domain between GAT and GAE. Previous studies have established that the VHS domain binds acidic cluster-dileucine (ACLL) sorting signals in the cytoplasmic domain of cargoes and that the GAT domain interacts with class I ARF GTPases that are essential for the recruitment of GGAs onto membranes. Moreover, an unstructured hinge region has been shown to bind clathrin, and the GAE domain reportedly binds a cohort of accessory proteins^5^. These domains mediate trans Golgi network (TGN)–endosome transport of cargo molecules with ACLL motifs, such as mannose 6-phosphate receptors (MPRs), sortilin, SorLA/LR11, LRP-3/9/12, BACE, stabilin-1, consortin, and Cl channel 7^5–7^. However, various *in vivo* roles and functional redundancies of GGAs and their cargoes remain poorly understood. Recent studies using knockout mice showed that deficiency of GGA2 but not GGA1 or GGA3 resulted in embryonic or neonatal lethality, indicating non-redundant functions of these three GGAs^6,8^. However, the specific roles of GGA2 in protein transport and the precise causes of death due to GGA2 deficiency are largely unknown. In this study, we determined the effects of GGA knockdown on EGFR trafficking and discovered a novel link between GGA2 and sustained EGFR expression that greatly supports the growth of cells.

## Results

### GGA2-depletion facilitates lysosomal degradation of EGFR via post-Golgi compartments

**Figure 1:**
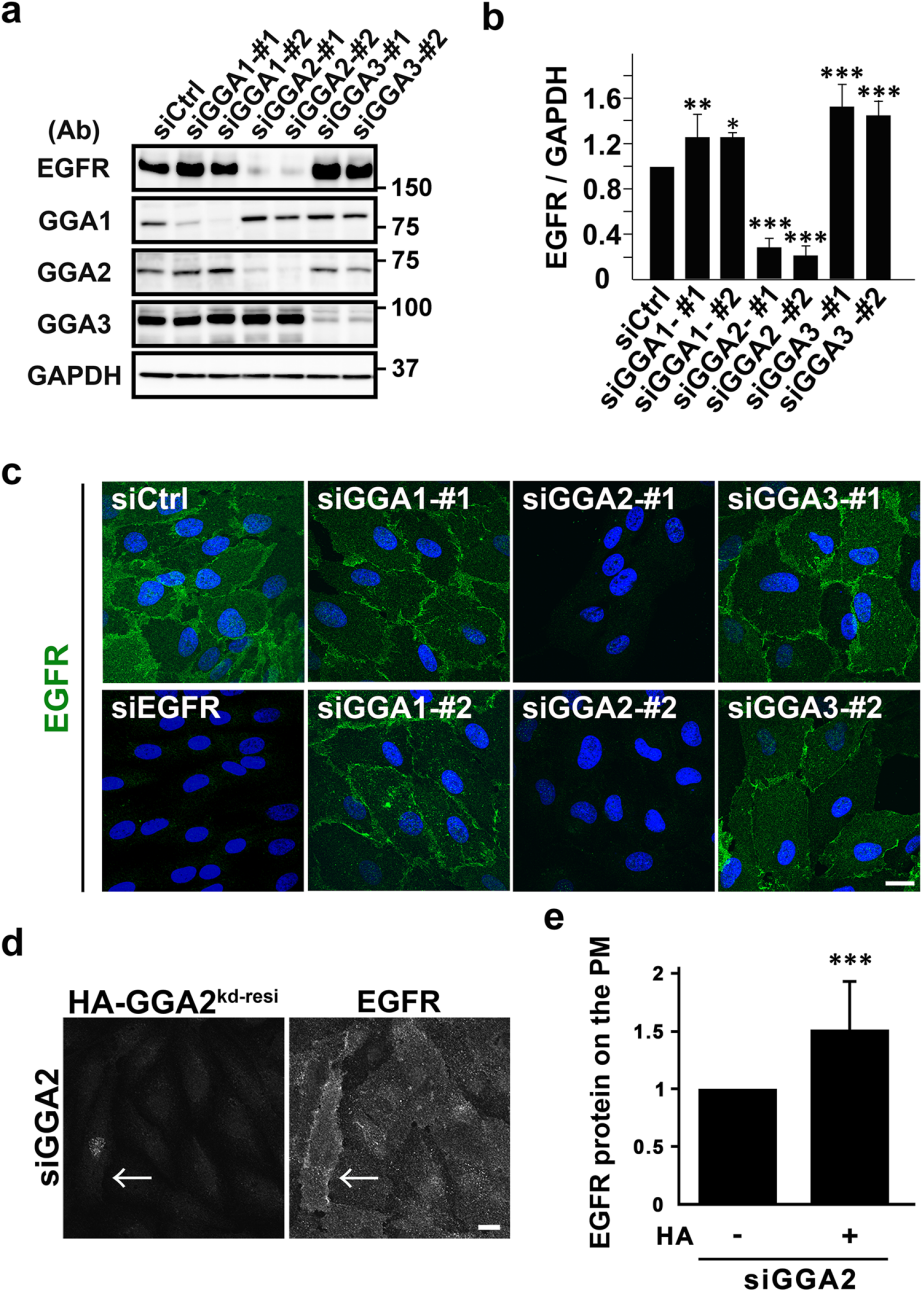
GGA2-depletion causes a drastic decrease in the EGFR protein expression. (**a**) Total lysates from ARPE-19 cells transfected with control (siCtrl), GGA1, GGA2, or GGA3 siRNAs were immunoblotted using indicated antibodies (Ab). Two sequences of siRNA (#1 and #2) were used for each GGA. Uncropped blots are presented in Supplementary Fig. 6. (**b**) Band intensities for EGFR were quantified in three or four experiments and mean values ± standard deviations (SD) were plotted. Differences between each GGA siRNA and siCtrl were analyzed by one-way ANOVA (*P* < 0.001) and Tukey’s honestly significant difference test (*P < 0.05, **P < 0.01, and ***P < 0.001). (**c**) Cells were treated with indicated siRNAs and were then fixed for immunofluorescence microscopy using anti-EGFR antibody. Nuclei were stained with Hoechst 33342 (blue); Bar, 20 μm. (**d**) GGA2-depleted cells re-expressing HA fused with siRNA-resistant full length GGA2 (HA-GGA2^kd-resi^) were double immunolabeled with anti-HA and -EGFR antibodies. Signal intensity for EGFR was enhanced to detect differences. The arrow indicates a cell expressing HA-GGA2^kd-resi^. Bar, 20 μm. (**e**) EGFR signal on the plasma membrane in HA-positive (n = 95) and -negative cells (n = 177) was quantified. Statistical analysis was performed using Student’s *t*-test (***P < 0.001). See Materials and Methods for details.

**Figure 2:**
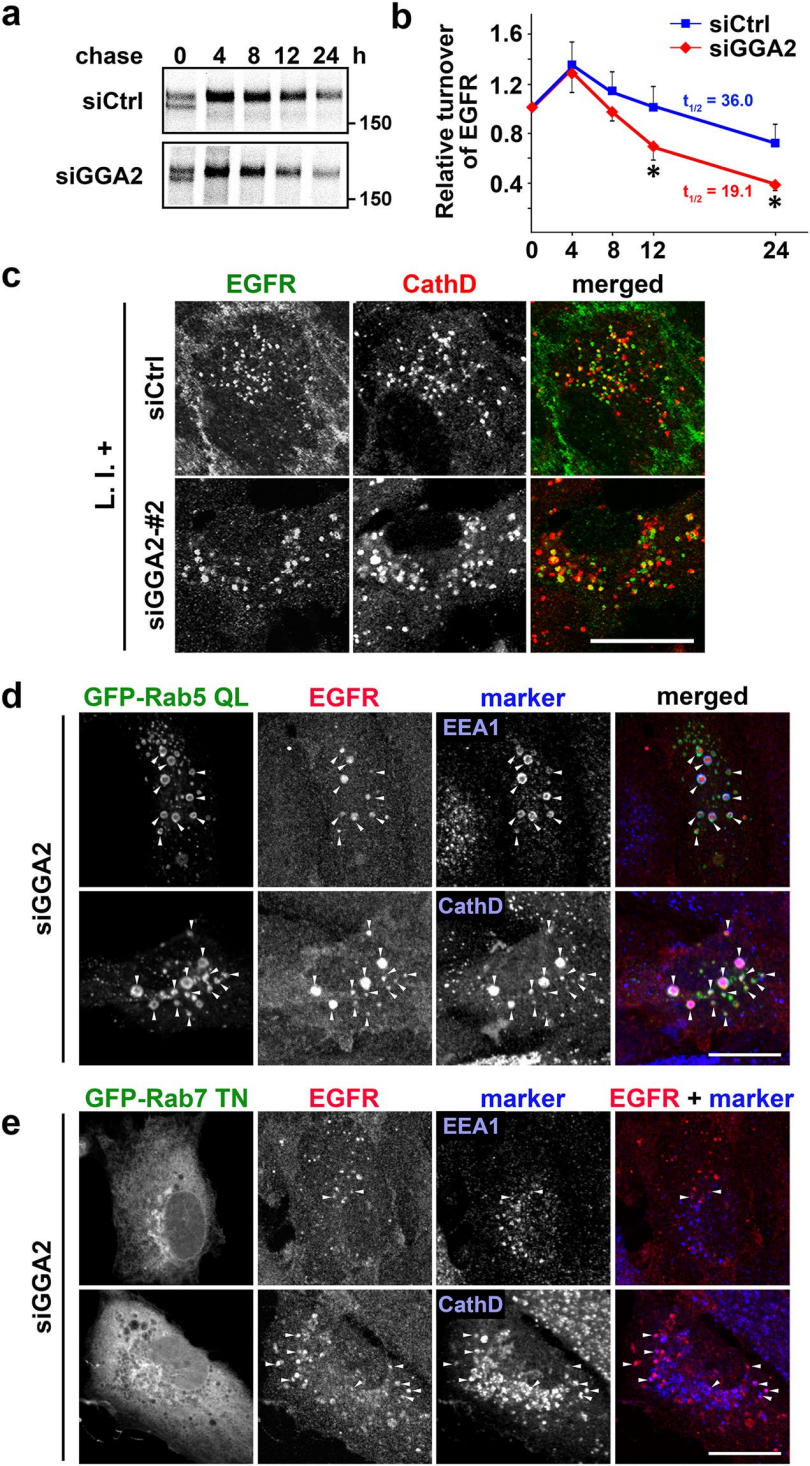
GGA2-depletion facilitates lysosomal degradation of EGFR via post-Golgi compartments. (**a**) Control (siCtrl) and GGA2-depleted (siGGA2) ARPE-19 cells were pulse-labeled with [^35^S]-methionine and cysteine, and were chased for indicated periods. The cell lysates were then subjected to immunoprecipitation using anti-EGFR antibody as described in Materials and Methods. Uncropped gels are presented in Supplementary Fig. 6. (**b**) Signals for EGFR were quantified in three experiments and were plotted as means ± SD. Differences between siCtrl and siGGA2 at each time point were identified using Student’s *t*-test; *P < 0.05. (**c**) Control (siCtrl) and GGA2-depleted (siGGA2-#2) ARPE-19 cells were treated with lysosomal protease inhibitors (L.I.+) for 6 h, followed by fixation and double immunofluorescence staining with anti-EGFR (green) and anti-cathepsin D (red) antibodies; Bar, 20 μm. See Supplementary Fig. S2a for images of low magnification and alternative siRNA for GGA2. (d and e) GGA2 knockdown ARPE-19 cells transfected with GFP-Rab5 QL (**d**) or GFP-Rab7 TN (**e**) were double immunostained with antibodies for EGFR and EEA1, or EGFR and cathepsin D (CathD). Arrowheads indicate colocalization of three signals (**d**), or two signals of EGFR and either EEA1 or cathepsin D (**e**). Bars, 20 μm.

To determine whether GGA depletion influences the behavior of EGFR, siRNAs with two specific target sequences for each GGA were applied to ARPE-19, a human retinal pigment epithelial cell line. EGFR protein expression in western blotting was greatly reduced following GGA2-depletion (*P* < 0.001, n = 4), and was slightly but significantly increased following GGA1- (*P* < 0.05, n = 3) or GGA3- (*P* < 0.001, n = 3) depletion (Fig. 1a and b). Subsequently, decreases in EGFR expression in GGA2-depleted cells were confirmed using immunofluorescence microscopy (Fig. 1c). Surface biotinylation experiments indicated that GGA2-depletion decreased EGFR expression not only in total lysates but also in the plasma membrane fractions (Supplementary Fig. S1a). Modest expression of HA-tagged GGA2 in GGA2-depleted cells partially restored EGFR localization to the plasma membrane (Fig. 1d and e), supporting that this effect was not caused by off-target effects of siRNAs. Moreover, pulse-chase experiments with [^35^S]-methionine and cysteine followed by immunoprecipitation of EGFR indicated increased turnover rates of EGFR in GGA2-depleted cells (Fig. 2a and b). Specifically, levels of EGFR mRNA and newly translated EGFR protein were unchanged after GGA2-depletion (Supplementary Fig. S1b–d). Moreover, treatment with lysosomal enzyme inhibitors increased a fraction of cytoplasmic punctate signals double positive for EGFR and cathepsin D, a lysosome marker, in both control and GGA2-depleted cells (Fig. 2c and Supplementary Fig. S2a and b). These results indicate that EGFR is posttranslationally missorted toward lysosomes for degradation in GGA2-depleted cells.

In further experiments, intracellular trafficking of EGFR to lysosomes was investigated in GGA2-depleted cells by expressing mutant proteins. Overexpression of GGA VHS-GAT reportedly inhibits ARF1-mediated function of GGAs, leading to retention of MPRs at the TGN^9,10^. Overexpression of VHS-GAT from GGA2 in GGA2-depleted cells increased EGFR expression, which was retained at perinuclear Golgi regions (Supplementary Fig. S2c). These data indicate that transport of EGFR from the ER to Golgi is not affected by GGA2 depletion. In subsequent experiments, endocytic pathways from early endosomes to late endosomes/lysosomes were perturbed by overexpressing mutant rabs. It has been shown that GFP-Rab5 QL overexpression causes a redistribution of EGFR into enlarged endosomal compartments that also contain markers for early/late endosomes and lysosomes^11–13^, and that overexpression of GFP-Rab7 TN impairs biogenesis of late endosomes/lysosomes^14,15^. EGFR expression was restored by concomitant expression of these mutant proteins in GGA2-depleted cells (Supplementary Fig. S2d and e). More precisely, EGFR was accumulated at abnormal early endosomal structures positive for both EEA1 and cathepsin D by GFP-Rab5 QL overexpression (Fig. 2d), or at aberrant late endosomal structures by GFP-Rab7 TN overexpression (Fig. 2e). Together, these results suggest that EGFR is constitutively transported between post-Golgi compartments before entering lysosomes for degradation in GGA2-depleted cells.

### GGA2 VHS-GAT recognizes EGFR juxtamembrane (jxt) region

**Figure 3:**
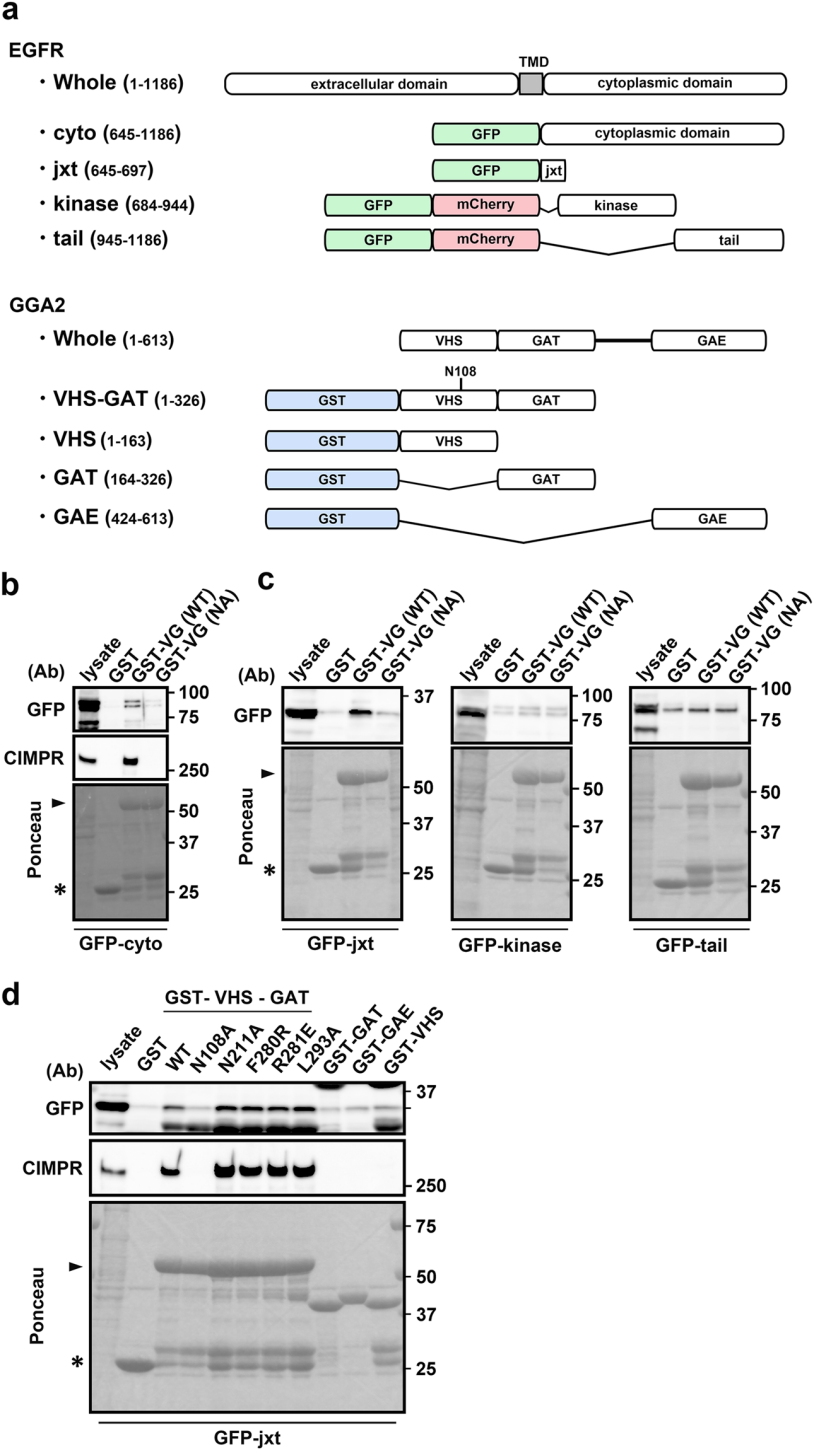
GGA2 VHS-GAT recognizes EGFR juxtamembrane (jxt) region. (**a**) Domain structures of EGFR and GGA2 mutants used in the binding assay are shown. Numbers indicate amino acid sequences of the proteins. N108A mutation site is indicated in VHS-GAT. TMD, transmembrane domain. (**b** and **c**) HEK293 cells were transfected with GFP tagged with entire cytoplasmic domain of EGFR (cyto), or with its jxt, kinase, or tail region. Cell lysates were mixed with glutathione sepharose and GST-GGA2 VHS-GAT (GST-VG[WT]) or its N108A mutant (GST-VG[NA]). Bound proteins were then eluted and analyzed using SDS-PAGE and immunoblotting with anti-GFP or -CIMPR (only for [b]) antibody as indicated (Ab). (**d**) Cell lysates from HEK293 cells expressing GFP-jxt were pulled down using GST fusion proteins containing wild type (WT) or N108A-, N211A-, F280R-, R281E, or L293A-mutated forms of GGA2 VHS-GAT, or with GST proteins fused with GAT, GAE, or the VHS region of GGA2. Bound proteins were immunoblotted using indicated antibodies (Ab). Ponceau stainings are shown at the bottom. Arrows and arrowheads indicate GST and GST-VHS-GAT, respectively. Uncropped blots are presented in Supplementary Fig. 6.

To explore interactions between GGA2 and the cytoplasmic domain of EGFR, we performed GST pull-down assays and observed significant interactions between GST-GGA2 VHS-GAT and the GFP-tagged cytoplasmic region of EGFR, and with endogenous CIMPR (Fig. 3b). Mutation of the Asn108 residue (N108A), which is required for binding to ACLL in cargoes, also reduced EGFR binding, indicating a dependency on the ACLL recognition site in GGA2 (Fig. 3b). To identify EGFR sites that are recognized by GGA2 VHS-GAT, we examined jxt, kinase, and tail domains of EGFR using GGA2 binding assays (for domain organizations, see Fig. 3a). Pull down experiments showed that only the jxt domain–GGA2 VHS-GAT interaction was N108 dependent (Fig. 3c). In separate experiments, each GAT, GAE, and VHS domain of GGA2 did not interact with the jxt domain (Fig. 3d), and the association of GGA2 VHS-GAT was not affected by the GAT mutations N211A, F280R, R281E, and L293A (Fig. 3d), which reportedly reduce binding of GGAs to ARF1, rabaptin5, phosphatidylinositol 4-phosphate (PI4P), and ubiquitin, respectively^10,16–18^. Thus, the VHS domain was required but not sufficient for this interaction. We assume that the addition of N-terminal residues of GAT domain to VHS would restore this binding, as has been reported for CIMPR^19^. Taken together, similar to other ACLL containing cargoes, EGFR jxt domain is recognized by the GGA2 VHS-GAT domain via interactions involving N108 in the VHS domain.

### GGA2 colocalizes and interacts with EGFR *in vivo*

**Figure 4:**
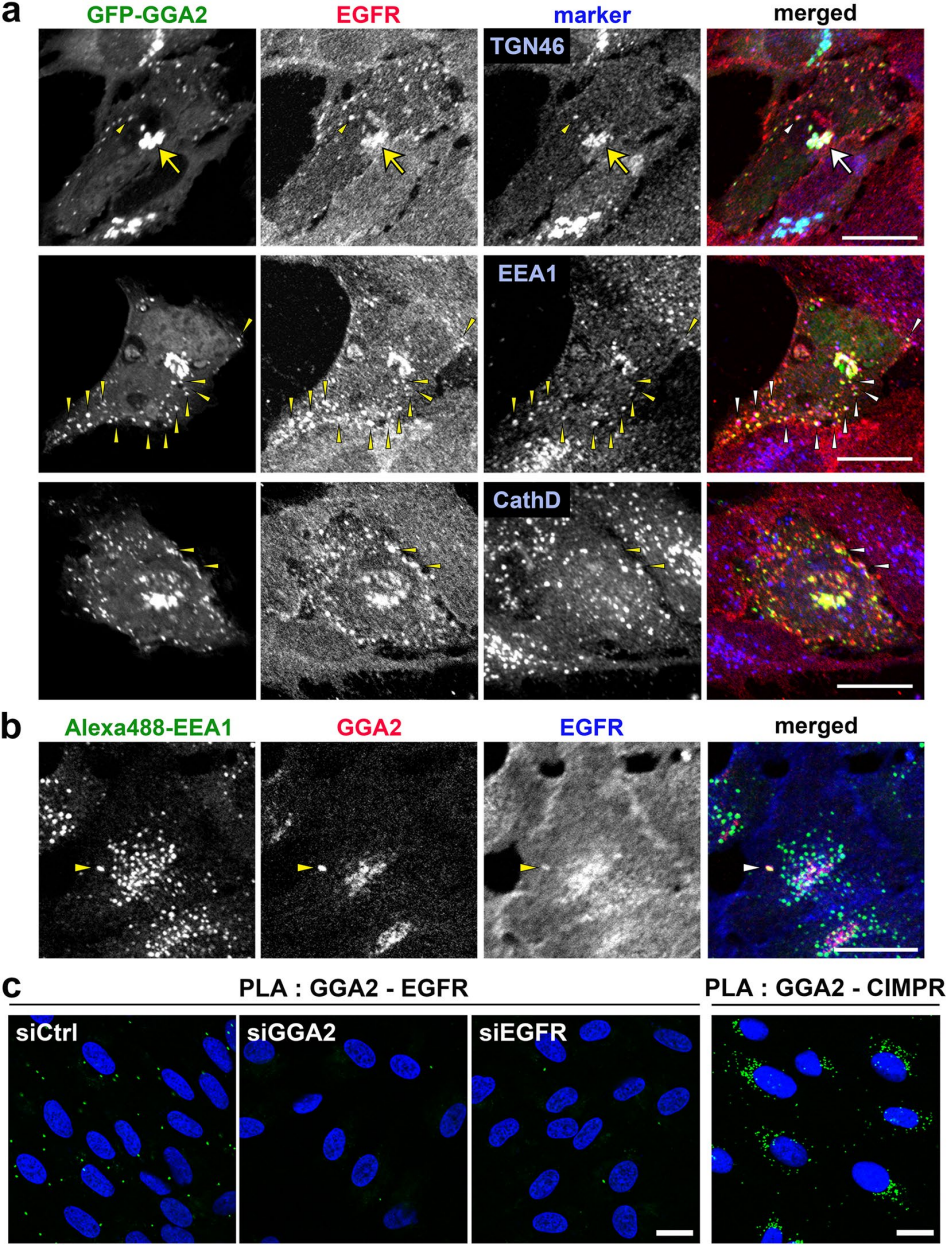
GGA2 colocalizes and interacts with EGFR *in vivo*. (**a**) ARPE-19 cells overexpressing GFP-tagged GGA2 were fixed and double immunofluorescence staining was performed using antibodies against EGFR (red) and TGN46, EEA1, or cathepsin D (blue). Arrows and arrowheads indicate colocalization of three signals in the TGN and peripheral structures, respectively. Bar, 20 μm. (**b**) ARPE-19 cells were fixed for triple immunofluorescence analyses using antibodies against Alexa488-EEA1 (green), endogenous GGA2 (red) and EGFR (blue). Arrowheads indicate colocalization of three signals. Bar, 20 μm. (**c**) ARPE-19 cells transfected with siCtrl, siGGA2, or siEGFR were processed for PLA to detect GGA2-EGFR or GGA2-CIMPR interaction (green). Nuclei were stained with DAPI (blue). Bars, 20 μm.

In triple labelling experiment, overexpressed GFP-GGA2 was colocalized with EGFR in some intracellular compartments, including the TGN, EEA1-positive early endosomes (58.4 ± 11%; n = 10) and cathepsin D-positive lysosomes (13.3 ± 11%; n = 10; Fig. 4a). Moreover, very small fractions of endogenous GGA2 colocalized with EGFR in the early endosomes (Fig. 4b). To corroborate interactions between GGA2 and EGFR *in situ*, we applied the proximity ligation assay (PLA)^20^ to immunofluorescence microscopy. Definite cytoplasmic signals for GGA2-EGFR interaction were detected, whereas they were drastically reduced by the knockdown of GGA2 or EGFR (Fig. 4c). This assay also revealed that the distribution pattern was largely different from perinuclear signals for GGA2-CIMPR interaction (Fig. 4c), suggesting that the GGA2-EGFR interaction mainly occurs in endosomal structures. These results indicate that GGA2 interacts with EGFR *in vivo*.

### GGA1 and GGA3 negatively regulate EGFR protein expression

**Figure 5:**
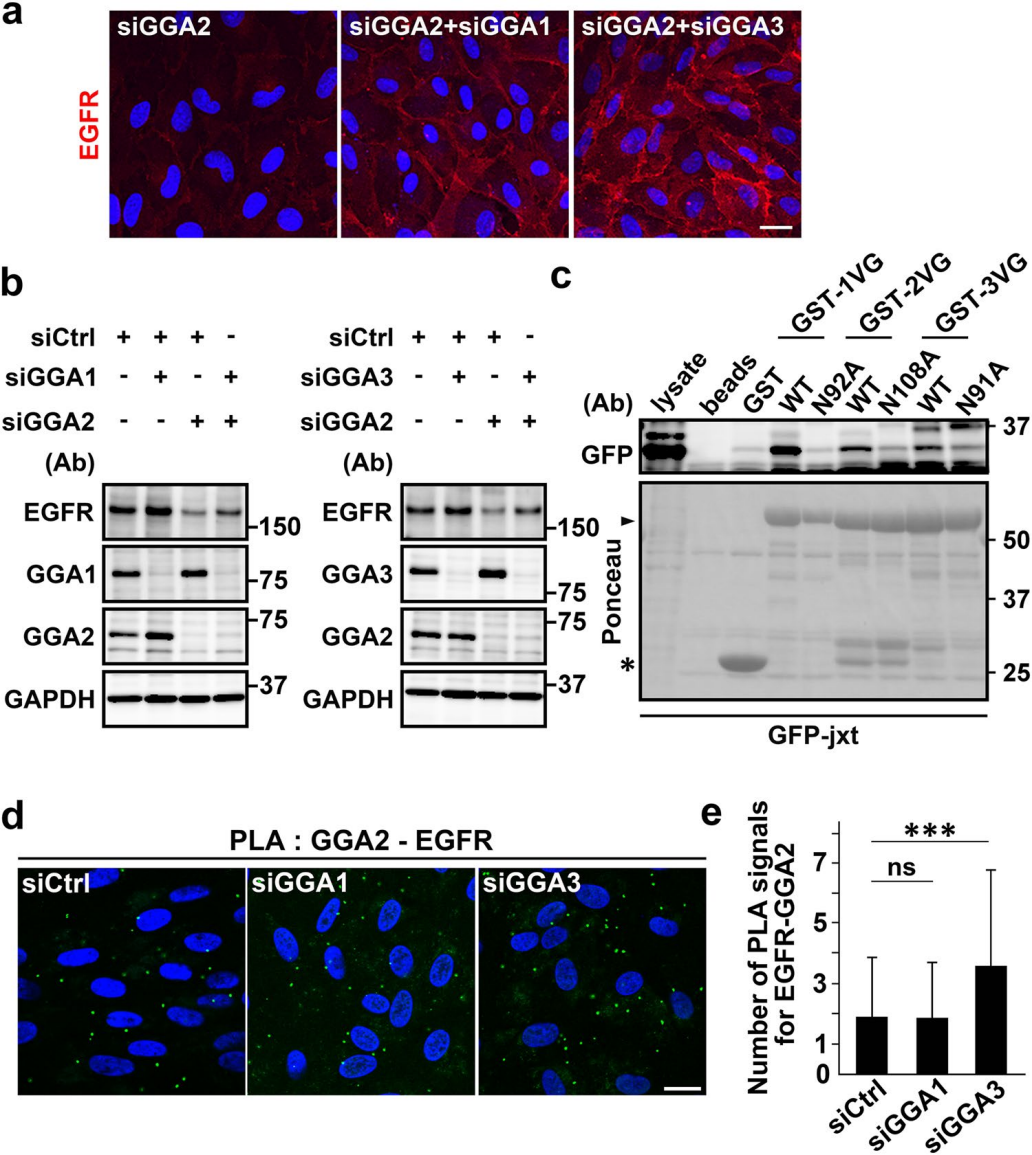
Simultaneous depletion of GGA1 or GGA3 with GGA2 restores EGFR expression. (**a** and **b**) ARPE-19 cells were transfected with control (siCtrl), GGA1 (siGGA1), GGA2 (siGGA2), or GGA3 (siGGA3) siRNAs alone, or with a combination of siGGA2 and siGGA1, or siGGA2 and siGGA3 as indicated. Cells were then fixed for immunofluorescence staining with anti-EGFR antibody (**a**), or were lysed for western blot analysis with antibodies against EGFR, GGA1, GGA2, GGA3, and GAPDH (**b**). Nuclei were labeled with Hoechst 33342. Bar, 20 μm. (**c**) Cell lysates from HEK293 cells expressing GFP-jxt were pulled down using GST fusion proteins containing wild type VHS-GAT (WT) of GGAs or mutant forms (N92A, N108A, or N91A) of VHS-GAT derived from GGA1 (GST-1VG), GGA2 (GST-2VG), or GGA3 (GST-3VG). Bound proteins were immunoblotted using anti-GFP antibody. Ponceau staining is shown at the bottom. Uncropped blots are presented in Supplementary Fig. 7. (**d**) ARPE-19 cells transfected with siCtrl, siGGA1, or siGGA3 were used for PLA to detect GGA2-EGFR interaction (green). Nuclei were stained with DAPI (blue). Bar, 20 μm. (**e**) PLA signals/cell were counted and mean values ± SD (n = 575 [siCtrl], n = 482 [siGGA1], and n = 448 [siGGA3]) were plotted. Differences were analyzed by one-way ANOVA (*P* < 0.001) and Tukey’s honestly significant difference test (***P < 0.001, ns: not significant).

Previously, GAT domains of GGA1 and GGA3 were shown to interact with ubiquitin^16,21,22^, and binding of GGA3 to ubiquitinated EGFR reportedly facilitated its lysosomal degradation^16^. Therefore, functions of GGA1 and GGA3 in GGA2-depleted cells may be augmented for EGFR degradation. In the present western blotting and immunofluorescence experiments, additional depletion of GGA1 or GGA3 restored EGFR expression in GGA2-depleted cells (Fig. 5a and b). Moreover, binding assays showed that the EGFR jxt domain was recognized by VHS-GAT from GGA1 and GGA3, and that the interactions were dependent on N92 and N91 residues, respectively, which are responsible for binding to ACLL motifs (Fig. 5c). In subsequent experiments, influences of GGA1- or GGA3-depletion on the GGA2-EGFR interaction were investigated using PLA. Knockdown of GGA3, but not of GGA1, caused a significant increase in the GGA2-EGFR interaction (Fig. 5d and e). These observations strongly suggest that the three GGAs can bind to the EGFR jxt domain, and that GGA1 and GGA3, but not GGA2, facilitate EGFR transport to lysosomes.

### GGA2-depletion suppresses cell growth *in vitro* and *in vivo*

**Figure 6:**
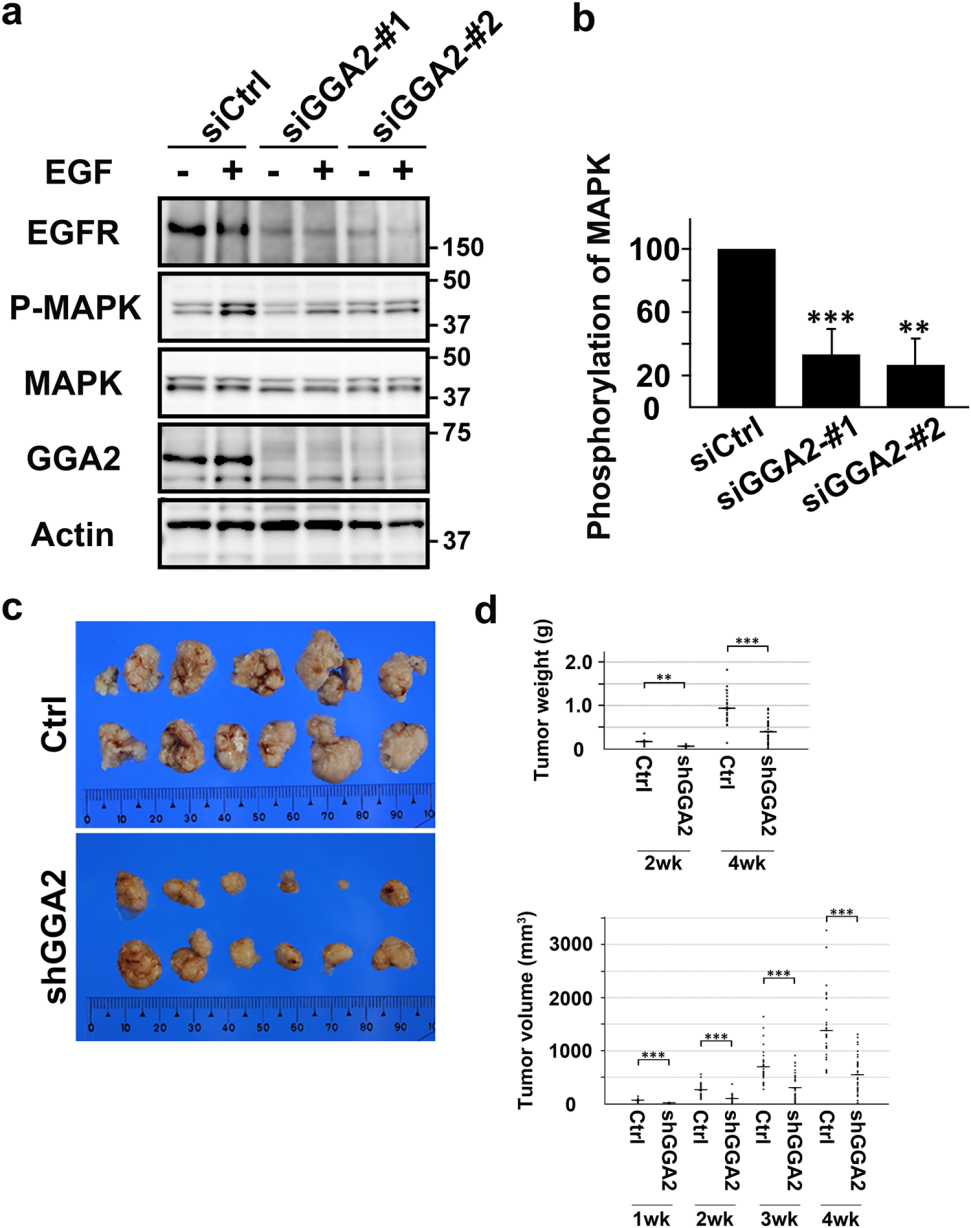
EGF-induced EGFR signaling and cell growth are impaired in GGA2-depleted cells. (**a**) Control (siCtrl) and GGA2-depleted (siGGA2-#1 and siGGA2-#2) ARPE-19 cells were treated with (+) or without (−) 10 nM EGF for 5 min and were then lysed for western blot analyses with antibodies against EGFR, phosphorylated MAPK (P-MAPK), MAPK, GGA2, or tubulin. Uncropped blots are presented in Supplementary Fig. 7. (**b**) Increase in the ratio of P-MAPK to MAPK after EGF stimulation was calculated and normalized to the value of siCtrl. Mean (±SD) of three experiments were plotted. Statistical difference between the control and each GGA2 siRNA was identified using Student’s *t*-test (**P < 0.01 and ***P < 0.001). (**c**) Control (Ctrl) or GGA2-depleted (shGGA2) LoVo cells were transplanted into nude mice. Tumors were excised at 4 weeks after transplantation. (**d**) Tumor weights and volumes were measured at the indicated time points and displayed as dot plots (mean [horizontal bar] ± SD). Statistical analyses were performed using Mann-Whitney *U*-test; ***P < 0.001.

In further investigations of the effects of GGA2-depletion on cell growth, downstream signaling of EGFR was monitored according to MAPK phosphorylation, which was significantly reduced in GGA2-depleted ARPE-19 cells (Fig. 6a and b). To examine cell growth, we selected A549, ARPE-19, HeLa, and LoVo cells, whose growth can be suppressed by an EGFR inhibitor, cetuximab (Supplementary Fig. S3a). shRNA-GGA2 was then introduced to generate cell lines that constitutively deplete GGA2. All of these cell lines showed decreased EGFR expression (Supplementary Fig. S3b) and impaired growth rates (Supplementary Fig. S3c). Finally, following application of LoVo cells in xenoplantation experiments, GGA2-depletion significantly suppressed growth rates of tumors (Fig. 6c and d). These results suggest that GGA2 controls cell growth by regulating EGFR expression.

### GGA2 protein is upregulated in human cancer tissues

**Figure 7:**
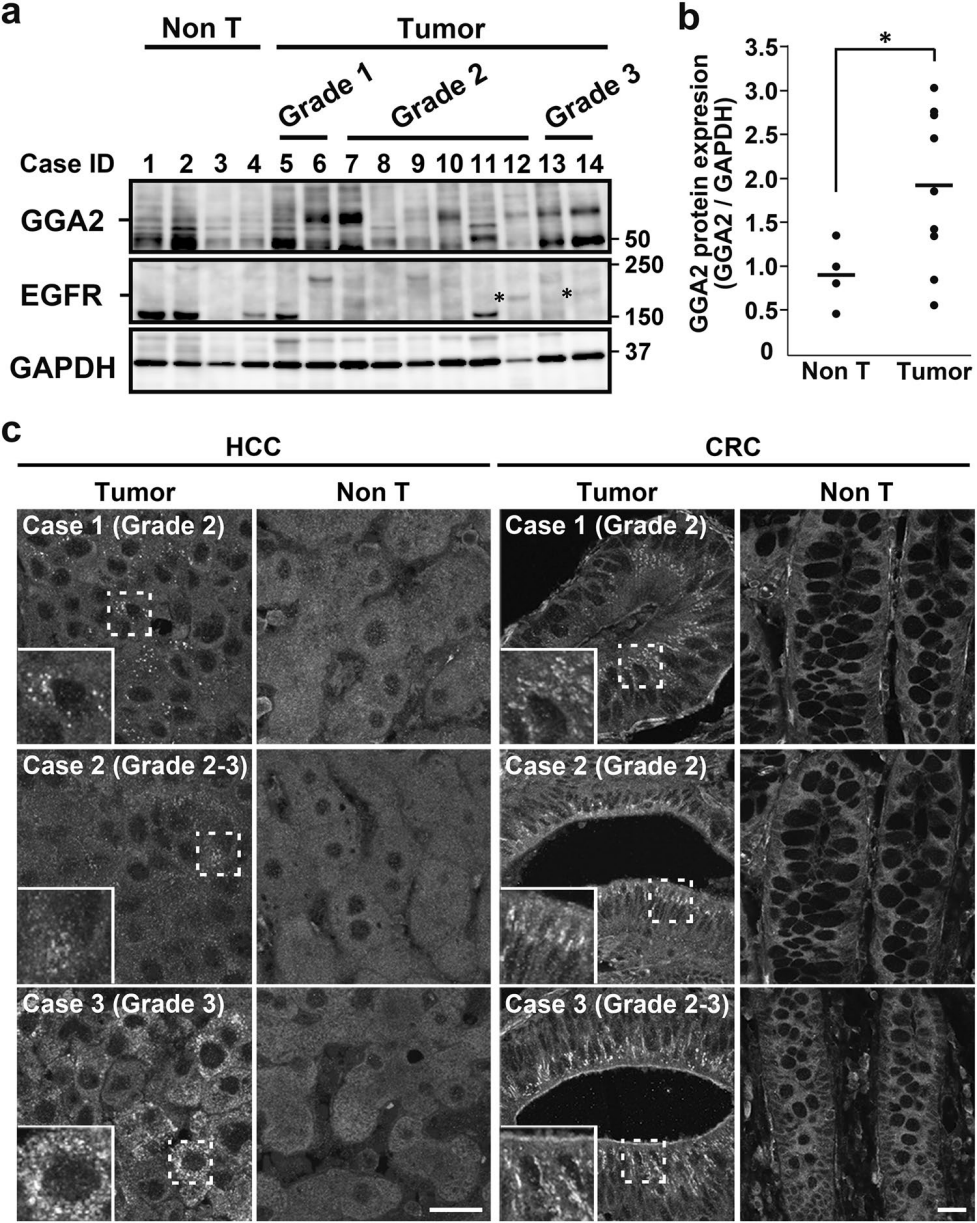
Expression of GGA2 in human hepatocellular carcinoma (HCC) and colorectal carcinomas (CRC). (**a**) Western blot analysis of GGA2 and EGFR expressions in non-tumor (Non T; 4 cases) and tumor regions of HCCs (10 cases). Asterisks indicate bands for EGFR. Histological grade is shown for each case (see Supplementary Table S1 for more detail). Uncropped blots are presented in Supplementary Fig. 7. (**b**) Ratios of GGA2 to GAPDH were calculated and normalized to that of case #1. Statistical analysis was performed using Mann-Whitney *U*-test; *P < 0.05. (**c**) Paraffin sections of tumors and adjacent non-tumor regions (Non T) from three cases of HCC and CRC were immunostained using anti-GGA2 antibody. Histological grade is shown for each case. Boxed regions are magnified and shown in insets; Bars, 20 μm.

In subsequent studies, we investigated the relationship between GGA2 expression and cancer using hepatocellular carcinoma (HCC) tissues. Western blot analyses showed that the GGA2 expression was significantly higher (*P* < 0.05, Mann-Whitney *U*-test) in tumor regions than in non-tumor regions (Fig. 7a and b). However, expression of GGA2 mRNA in corresponding samples did not necessarily increase (Supplementary Fig. S4c). Interestingly, higher expressions of EGFR protein were observed in 2 (#12 and 14) out of 6 cases that showed apparently higher expressions of GGA2 (Fig. 7a). Similarly, expressions of both GGA1 and GGA3 were higher in some tumor regions than in non-tumor regions, but the increases were not statistically significant (Supplementary Fig. S4a and b). Also, mRNA expressions of GGA1 and GGA3 did not correlate with the protein expression levels (Supplementary Fig. S4c). Tissue arrays of HCC and colorectal carcinoma (CRC) were then examined by immunohistofluorescence microscopy using specific antibody against GGA2 (Supplementary Fig. S5). As shown in Fig. 7c several cytoplasmic punctate signals for GGA2 were detected in 30.8% (4/13 cases) of HCC and 23.3% (7/30 cases) of CRC cases. These results indicate that higher GGA2 protein expression may support cell growth in significant proportions of HCC and CRC tissues.

## Discussion

The relationship between GGAs and EGFR has been reported for GGA3, which links ubiquitinated EGFR with ubiquitin sorting machinery at endosomes^16^. In this study, reduced expression of GGA3 resulted in accumulation of endocytosed EGFR within enlarged endosomes. Although we partly confirmed this observation by demonstrating that GGA3 or GGA1 depletion increased cellular EGFR expression, EGFR expression was drastically reduced following GGA2 depletion. To our knowledge, these are the first data to show opposing effects of GGA2 and GGA1/3. Moreover, the present experiments show that all three GGAs interact with the EGFR jxt domain, and that these interactions are dependent on the amino acid that is responsible for recognition of ACLL signals in cargoes. Previous studies have shown that the EGFR jxt domain contains a di-leucine motif at the 679/680 (679-LL) position, which is required for efficient lysosomal degradation of EGFR^23,24^. However, 679-LL does not conform to the consensus ACLL signal (DXXLL) for GGAs due to the lack of an N-terminal acidic residue. Moreover, NMR spectroscopy revealed α-helical structural propensities of these residues, which is distinguished from ACLL that are recognized in extended β or β-like conformations^25^. Therefore, although roles of 679-LL in the interaction of GGAs with EGFR jxt have not been shown, the binding mode may differ from known mechanisms, warranting further studies of additional sites that are involved in this interaction.

Multiple studies have demonstrated that GGA1/3 but not GGA2 recognize ubiquitin^16,21,22^. Moreover, using crystal structure analyses, Prag *et al*.^26^ showed sequence differences in ubiquitin-binding hydrophobic patches on GAT domains of GGA1/3 and GGA2, potentially reflecting the lower affinity of GGA2 than that of GGA1/3. In the present study, EGFR expression in GGA2-depleted cells was restored by additional knockdown of GGA1 or GGA3. Hence, although all three GGAs bind to EGFR jxt domains, GGA1/3 but not GGA2 mediates EGFR degradation. Moreover, *in situ* PLA apparently demonstrated that GGA3 depletion, but unfortunately not GGA1 depletion, caused increased signal for GGA2-EGFR interaction. Accordingly, these observations suggest that GGA1/3 and GGA2 have an opposing function for EGFR degradation via their common binding site in EGFR. Interestingly, this mechanism might proceed under no additional EGF stimulus, which is in contrast to the GGA3-mediated degradation of ubiquitinated EGFR occurring after administration of EGF^16^. However, it may be possible that the binding of GGA1/3 to EGFR jxt domain contributes to the efficient associations between GGA1/3 and ubiquitin on EGFR at steady-state for its lysosomal degradation. Other properties that distinguish GGA2 from GGA1/3 have been reported. Among these, GGA2 lacks the autoinhibitory mechanisms that are mediated by interactions between VHS and ACLL in the hinge^27,28^ and GGA2 is more efficiently enriched in isolated HeLa clathrin coated vesicles than GGA1/3^29,30^. Although these differences can be applied to the ACLL-containing cargoes, it may somehow link to the interaction between GGA and EGFR jxt domain. It should also be noted that GGA1 appeared to bind the jxt domain with highest affinities among GGAs (Fig. 5C), while depletion of GGA3, but not GGA1, affected the PLA signal for GGA2-EGFR interaction (Fig. 5d and e). Although these results may suggest different behaviors between GGA1 and GGA3 for the binding to jxt domain *in vitro* and *in vivo*, the precise mechanisms remain elusive. Moreover, compelling evidence of distinguishing features of GGAs has been shown using GGA-deficient mice^6,8^. According to these studies, GGA2-deficient mice are embryonic or neonatal lethal, whereas GGA1- or GGA3-deficient mice lack severe phenotypes. This phenotypic difference may be consistent with the opposing actions of GGA2 and GGA1/3 on EGFR stabilization. Potentially, the lack of EGFR and its signaling cascades in GGA2-deficient embryos and neonates would arrest development, although the ensuing details remain subjects of future studies.

To identify sites of GGA2-affected EGFR trafficking, we demonstrated occasional colocalization of endogenous GGA2 and EGFR in early endosomes by immunofluorescence, and *in situ* interaction of both molecules by PLA. The PLA data also indicated that the interaction occurred preferentially in the peripheral area rather than the perinuclear TGN in cells. Moreover, EGFR was successfully trafficked to the endocytic pathway in GGA2-depleted cells, as indicated by blocked receptor transport in experiments using mutants for Rab5 and Rab7. Taken with a previous study showing that GGA3 promoted degradation of ubiquitinated EGFR in MVBs^16^, the present data indicate that GGA2 together with GGA1/3 might regulate EGFR trafficking in early endosomes/MVBs. Nonetheless, these lines of evidence do not completely exclude the possibility that GGA2-depletion affects other trafficking machineries. More recently, it has been shown that GGA3 promotes recycling of another RTK, Met, from endosomes in association with gyrating clathrin structures^31,32^. It is still an open question if this dynamic structures would be involved in the GGA2-mediated EGFR stabilization.

Finally, we demonstrated GGA2 expression is required for cell growth *in vitro* and in a xenograft model, and that a significant proportion of HCC (~31%) and CRC cells (~23%) showed higher levels of GGA2 expression. In agreement, overexpression of EGFR protein has been reported in 40–70% of conventional HCC and 35–50% of CRC patients^33–35^. However, increased EGFR protein expression did not correlate with oncogenic mutations or increased EGFR copy numbers in previous studies^33,36^. Therefore, EGFR overexpression may reflect dysregulation of EGFR protein turnover, among other mechanisms. The present results suggest that EGFR is stabilized in cancer cells that overexpress GGA2, likely supporting growth of HCC and CRC. However, because the present data also indicated that the protein expressions of GGA1 and/or GGA3 were also increased in some cancer tissues, relative amounts of GGA1, 2, and 3 could determine the EGFR stability. Further studies with increasing number of patient cases are required to determine correlations between EGFR and three GGAs in cancer tissues. In our searches of previous DNA microarray analyses, GGA2 was not listed as a significantly upregulated gene in HCC^37–43^ or CRC^44–49^. In agreement with this, our data indicated no correlation of GGA2 protein expression with mRNA expression. Therefore, GGA expression may be primarily regulated by protein turnover mechanisms.

To date, some of GGA cargos containing ACLL motifs have also been involved in tumor growth. CIMPR is also known as the IGF2 receptor suppressing cell growth by IGF2 clearance^50^. Sortilin has recently been reported to promote EGFR internalization limiting its signaling^51^. It remains unclear how these mechanisms would affect GGA-mediated EGFR transport and tumor growth *in vivo*. Taken together, we conclude that GGA2-assisted stabilization of EGFR contributes to the growth of cells, offering a potential therapeutic target for anticancer drug development.

## Materials and Methods

### Antibodies and reagents

Experiments for protein immunodetection were performed using the following antibodies: rabbit antibodies against EGFR (Cell Signaling Technology [4267] and Santa Cruz Biotechnology [sc-03]), GGA1 (Santa Cruz Biotechnology [sc-30102]), p44/42 MAP kinase (Cell Signaling Technology [4695]), p-p44/42 MAP kinase (Cell Signaling Technology [9101]), EEA1 (Cell Signaling Technology [2411]), CIMPR (Epitomics [5230-1]), and Alexa488-EEA1 (MBL [M176-A48]); mouse antibodies against EGFR (Millipore [05-101]), GAPDH (Santa Cruz Biotechnology [sc-32233]), β actin (Santa Cruz Biotechnology [sc-47778]), GGA2 (BD Transduction Laboratories [612612]), GGA3 (BD Transduction Laboratories [612310]), and GFP (Roche [11814460001]); a rat antibody against HA (Roche [11867423001]); a sheep antibody against TGN46 (Serotec [AHP500GT]). Immunohistofluorescence analyses of GGA2 were performed in paraffin-embedded tissues using an anti-GGA2 antibody that was generated by immunizing rabbits with a synthetic peptide carrying the sequence QNPSADRNLL, which lies within the hinge domain of human GGA2. The specificities of this antibody were demonstrated in Supplementary Fig. S5.

Cetuximab (Erbitux) were purchased from MERCK. E64d and Leupeptin were purchased from Peptide Institute, Inc.

### Constructs

Polymerase chain reactions (PCR) were performed using PrimeSTAR or PrimeSTAR MAX (Takara). Mutations were introduced using a QuikChange site-directed mutagenesis kit (Stratagene). GFP-Rab5 QL and GFP-Rab7 TN constructs were gifts from Mitsunori Fukuda (Tohoku University, Japan)^52^.

### Knockdown by siRNA and shRNA

Target sequences for siRNAs were as follows:

human GGA1-#1 (5′-CUGGAGGCGCGAAUCAAUAGA-3′),

human GGA1-#2 (5′-GGCAAGUUCCGCUUUCUCAAC-3′),

human GGA2-#1 (5′-GGUUUCCGGAAGACAUCAAGA-3′),

human GGA2-#2 (5′-GGAGUUCUGCUGUACAAACAG-3′),

human GGA3-#1 (5′-CUGCAGUGCCCAAGUCAAUGA-3′),

human GGA3-#2 (5′-GCUGCAGUGCCCAAGUCAAUG-3′),

human EGFR (ON-TARGET plus SMARTpool (Thermo Scientific).

Unless otherwise indicated, GGA1-#2, GGA2-#2, and GGA3-#1 were used in GGA-knockdown experiments. Cells were transfected with siRNAs using Lipofectamine RNAiMAX (Invitrogen), and were processed for immunostaining or western blotting 3 days later. For shRNA experiments, a pLKO.1 puro vector (Sigma-Aldrich) that contains GGA2 shRNA (corresponding to the GGA2-#2 target sequence) was constructed according to the manufacturer’s instructions. Subsequently, HEK293T cells were transfected with GGA2 shRNA vector or pLKO.1 empty vector as a control for 24 h, and virus-containing media were then collected. Cells were infected with the lentivirus in DMEM containing 10% FBS and 8 µg/ml polybrene for 20–24 h, and media were then replaced with fresh media containing puromycin at concentrations of 1–2.5 µg/ml. Following confirmation of knockdown efficiency in western blotting experiments, the bulk of puromycin-resistant cells were used for *in vitro* cell growth assays and xenotransplantation.

### Western blot analysis

Cells were lysed in PBS containing 1% Triton X-100 and protease inhibitor cocktail (Roche). Lysates were then separated on 10% gels or 5–20% gradient gels (Wako), and were transferred to PVDF membranes (Millipore). After blocking with 10% skim milk in 1 × TPBS, membranes were incubated with the primary antibodies described above, and bound antibodies were detected using ECL Prime (GE Healthcare) and ImageQuant LAS4000 mini (GE Healthcare) software. Band signal intensities were quantified using ImageJ. In EGF stimulation experiments, cells were incubated in DMEM containing 0.1% BSA for 20 h, and were then incubated with 10 nM EGF in the same media for 5 min. Subsequently, cells were lysed in 1 × SDS-PAGE sample buffer and were processed for western blot analyses.

### GST pull-down experiments

GST pull-down experiments were performed following transfections of HEK293T cells with various plasmids. Cells were lysed in 50 mM HEPES buffer (pH 7.3) containing 5 mM EDTA, 100 mM NaCl, 1% Triton X-100, and a protease inhibitor cocktail (Roche). After pre-incubation with 10 µg of GST-fusion protein and glutathione-conjugated sepharose beads for 16–20 h at 4 °C, lysates were added and were further incubated for 16–20 h at 4 °C. Beads were then washed three times in lysis buffer, and bound proteins were eluted from the beads by boiling for 10 min in 1 × SDS-PAGE sample buffer at 90 °C. Approximately 5% of input was applied to the gel lane labeled with “lysate” in Figures.

### Pulse-chase analysis and immunoprecipitaiton

Cells were incubated in DMEM without methionine or cysteine (Met/Cys [-]) for 30–60 min, and in DMEM (Met/Cys [-]) containing a mixture of [^35^S] methionine and cysteine (Perkin Elmer) for 120 min. Cells were then washed three times in cold DMEM and were incubated in DMEM containing 10% FBS for 0, 4, 8, 12, and 24 h. They were lysed in PBS containing 1% Triton X-100 and protease inhibitor cocktail, and the lysates were incubated with Protein A-conjugated sepharose beads for 1 h and then collected in new tubes. Lysates were incubated with 2 µg of anti-EGFR antibody (Millipore) for 20–24 h and beads were then washed five times in lysis buffer. Bound proteins were then eluted from the beads with 1 × SDS-PAGE sample buffer followed by boiling at 90 °C for 10 min, and were finally analyzed using SDS-PAGE. Dried gels were exposed to a BAS-imaging plate and were scanned using a BAS2500 phosphorimager (Fujifilm). EGFR band intensities were quantified using a software of Multi Gauge Ver2.02 (Fujifilm) or ImageJ. All experiments were repeated three times.

### Surface biotinylation assay

ARPE-19 cells transfected with siCtrl, siGGA2-#1 or siGGA2-#2 were washed with ice-cold PBS 3 times, and were incubated with PBS containing 0.5 mM EZ-Link Sulfo-NHS-SS-biotin (ThermoFisher [29129]) for 30 min on ice. Then, they were incubated with ice-cold TBS buffer (10 mM Tris-HCl [pH7.5], 150 mM NaCl) on ice for 15 min, and were lysed with TBS buffer containing 1% Triton X-100 and protease inhibitor cocktail. Lysates were incubated with Avidin-agarose (ThermoFisher [20228]) for 6 h. They were washed 5 times using TBS buffer containing 1% Triton X-100, and bound proteins were eluted by boiling 10 min in 1 × SDS-PAGE sample buffer at 90 °C. Approximately 10% of input was applied to the gel lane labeled with “total” in Figures.

### Immunofluorescence microscopy

Immunofluorescence microscopy was performed using standard protocols. Briefly, cells were fixed with 4% PFA in 0.1 M phosphate buffer (pH 7.4) and were then permeabilized in PBS containing 0.1% Triton X-100 and 0.4% BSA or in PBS containing 50 µg/ml digitonin and 0.4% BSA. They were incubated with primary antibodies followed by the appropriate secondary antibodies conjugated with Alexa488, Alexa594, or Alexa647 (Molecular Probes). Fluorescence images were obtained using a confocal microscope (FV1000, Olympus) equipped with a PlanApo N 60× (NA 1.42 oil) and a UPlan FL N 40× (NA 1.3 oil) lenses. To inhibit lysosomal degradation, cells were transfected with siRNAs and treated with E64d and Leupeptin for 6 h and were then fixed for immunofluorescence analyses. Percentages of double positive puncta for EGFR and cathepsin D over cathepsin D positive ones were measured.

For quantification of EGFR signal in GGA2-depleted ARPE-19 cells re-expressing HA-GGA2^kd-resi^, fixed cells were immunostained with anti-EGFR antibody (Millipore) without permeabilization, and then immunostained with anti-HA antibody following permeabilization. Four serial *x*-*y* images along *z*-axis, which cover majority of a whole cell, were acquired and projected into a single image. Mean intensity for EGFR signal per cell was quantified using ImageJ software.

### Analysis of cell growth *in vitro*

Cell lines were seeded on 96-well plates in triplicate at 1000–6000 cells per well. Cells were allowed to grow for at least 20 h, and were then incubated for the indicated number of days. Cell growth and viability assays were performed using Cell Counting Kit-8 (Dojindo) according to the manufacturer’s instructions.

### RNA isolation and reverse transcription-quantitative PCR

Total RNA was extracted from cells using ISOGEN according to the manufacturer’s instructions (Nippon Gene). First strand cDNAs were synthesized using SuperScript III First-Strand Synthesis SuperMix (Invitrogen) and quantitative PCR was conducted using TaqMan Gene Expression Assays (Applied Biosystems; EGFR: Hs01076078_m1, ACTB: Hs01060665_g1).

### Proximity ligation assay

Proximity ligation assay (PLA) was conducted using a PLA kit (DuolinkⓇ *In Situ*, Sigma) according to the manufacturer’s instructions. Antibodies used for this assay were as follows; rabbit anti-EGFR (Cell Signaling Technology [4267]), rabbit anti-CIMPR (Epitomics [5230-1]), and mouse anti-GGA2 (BD Transduction Laboratories [612612]). Nuclear staining was done using mounting medium with DAPI. Although red fluorophore-containing detection regent was used, the signal was assigned as a green color for better recognition in figures.

### Determination of transplanted tumor sizes

Four-week-old female BALB/c nu/nu nude mice were purchased from CLEA Japan. LoVo cells were stably transfected with control or GGA2 shRNA and were cultured. Cell suspensions were adjusted to 1.2 × 10^6^ cells per 0.1 ml, and were subcutaneously injected into the right and left sides of the backs of mice. Tumor sizes were measured weekly for 4 weeks and tumor volumes were calculated according to the following formula: *V* = *ab*^2^/2, where “*a*” and “*b*” represent the length and width of the xenograft, respectively. This study was approved by the Ethics Committee for Animal Research of Fukushima Medical University (approval number: 26015 and 24035). All animal experiments were performed in accordance with the guidelines and regulations of Fukushima Medical University.

### Analysis of human samples

Glass slides with tissue arrays of hepatocellular carcinoma (13 cases, DgLiv-02-005) and colon adenocarcinoma (30 cases, COLADE060) were purchased from Shanghai Outdo Biotech Co. After deparaffination and rehydration, tissue sections were prepared for immunohistofluorescence analyses using conventional methods. Briefly, antigen retrieval was performed using a microwave processor (Azumaya Corporation, Tokyo, Japan) in the solution Immunosaver (Nisshin EM Co., Ltd) for 20 min at 98 °C. Histological slides were then blocked and incubated for 3 days at 4 °C with the primary antibody against GGA2. For absorption test, the anti-GGA2 antibody was preincubated with GST or GST-fused with the hinge region of GGA2 (aa327-423) for 16 h at 4 °C, which were then applied to the HCC sections for 3 days at 4 °C. Protein lysates and Total RNA samples of HCC and control liver tissues were purchased from OriGene Technologies, Inc (see Supplementary Table S1). Following synthesis of first strand cDNA using SuperScript III First-Strand Synthesis SuperMix (Invitrogen), qRT-PCR was performed using TaqMan Gene Expression Assay (GGA1: Hs01081687_m1, GGA2: Hs00370910_m1, GGA3: Hs01597827_m1, ACTB: Hs01060665_g1) and TaqMan Gene Expression Master Mix (Applied Biosystems). This study was approved by the Institutional Review Board in Fukushima Medical University (approval number: 2943). All experiments using human samples were performed in accordance with the guidelines and regulations of Fukushima Medical University. For each human sample, informed consent was properly obtained as stated by the supplier companies (http://www.origene.com/Tissue/Tissue_QC.aspx; http://www.outdobiotech.com/en/biobankservices.html).

### Statistical analyses

For comparisons between three or more groups, one-way ANOVA followed by Tukey’s honestly significant difference test was applied. Data of xenograft experiments and of human samples were analyzed by Mann-Whitney *U*-test. These analyses were performed using a software, SPSS (IBM, Armonk, NY, USA). For others, Student’s *t*-test was used. For each analysis, *P* < 0.05 was considered statistically significant.

### Data availability

The datasets generated during the current study are available from the corresponding author on reasonable request.

## Electronic supplementary material

Supplementary information
