# Author Correction: GGA2 interacts with EGFR cytoplasmic domain to stabilize the receptor expression and promote cell growth

**DOI:** 10.1038/s41598-020-64604-1

**Published:** 2020-05-01

**Authors:** Takefumi Uemura, Satoshi Kametaka, Satoshi Waguri

**Affiliations:** 1 Department of Anatomy and Histology, Fukushima Medical University School of Medicine, 1 Hikariga-oka, Fukushima City, Fukushima 960-1295 Japan; 2 Department of Physical Therapy, Nagoya University Graduate School of Medicine, 1-1-20 Daiko-minami, Higashi, Nagoya City, Aichi 461-8673 Japan

Correction to: *Scientific Reports* 10.1038/s41598-018-19542-4, published online 22 January 2018

This Article contains an error. In Figure 7C, the panel for the adjacent non-tumor region (Non T) in colorectal carcinoma, case 1, is incorrect. The correct Figure 7 appears below as Figure 1.

**Figure 1:**
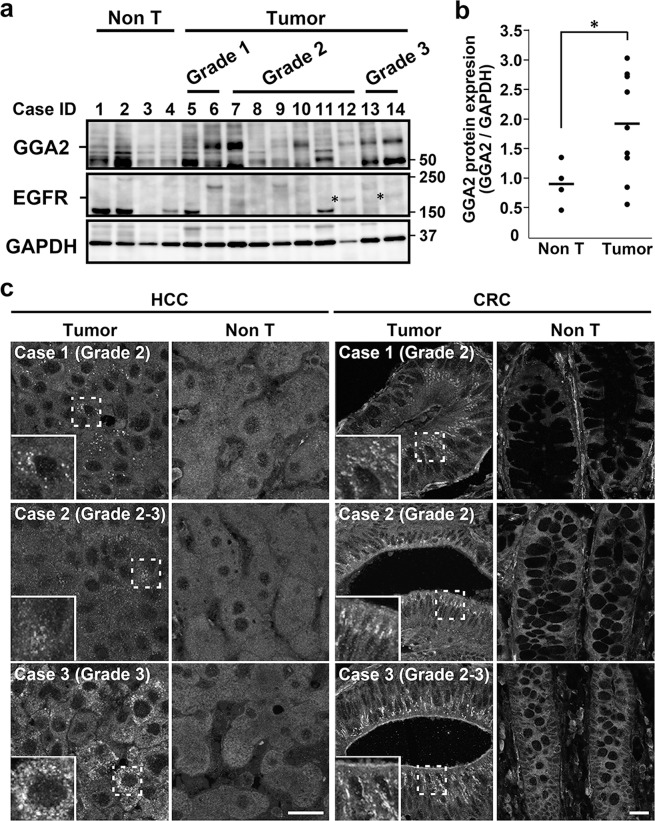
.

