# Supplementary material for: GGA2 interacts with EGFR cytoplasmic domain to stabilize the receptor expression and promote cell growth
Source: Sci Rep. 2018 Jan 22;8:1368. doi: 10.1038/s41598-018-19542-4 (PMC5778047; doi:10.1038/s41598-018-19542-4)
Supplement: Supplementary file 1 — Supplementary information [file 41598_2018_19542_MOESM1_ESM.pdf]

# - Supplementary information (Figures and Table) -

GGA2 interacts with EGFR cytoplasmic domain to stabilize the receptor expression and promote cell growth

Takefumi Uemura[1], Satoshi Kametaka[2], and Satoshi Waguri[1]*

[1]Department of Anatomy and Histology, Fukushima Medical University School of Medicine, 1 Hikariga-oka, Fukushima City, Fukushima 960-1295, Japan

[2]Department of Physical Therapy, Nagoya University Graduate School of Medicine, 1-1-20 Daiko-minami, Higashi, Nagoya City, Aichi 461-8673, Japan

Supplementary Fig. S1

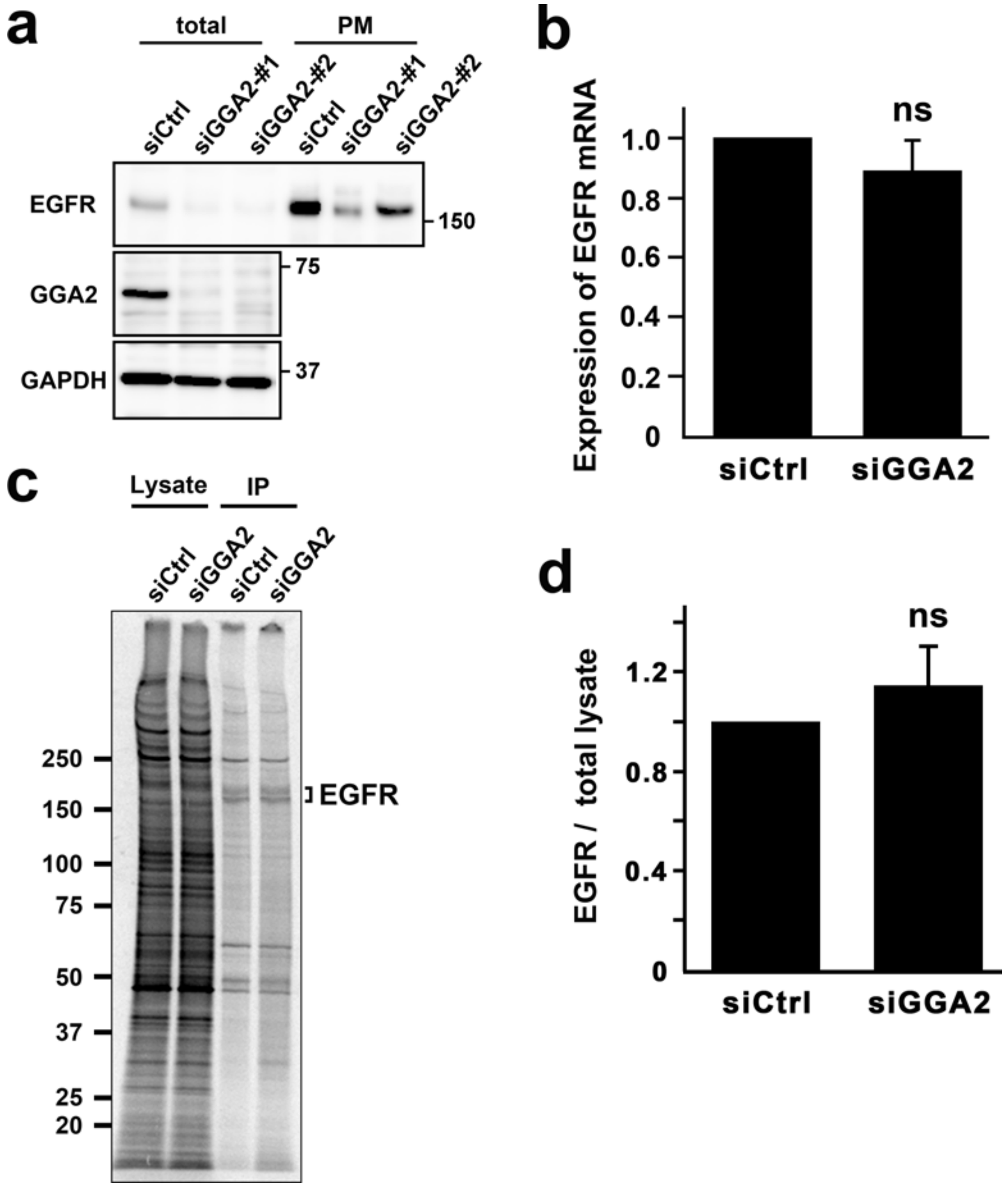

Supplementary Fig. S1. GGA2-depletion decreases protein expression of EGFR, but not affect its mRNA expression and transcription

(a) ARPE-19 cells transfected with control (siCtrl) or GGA2 (siGGA2-#1, -#2) siRNA were incubated with PBS containing 0.5 mM Sulfo-NHS-SS-biotin followed by lysis. Lysates were precipitated with Avidin-agarose. Approximately 10% of input was applied to the gel lane labeled with “total”. (b) EGFR mRNA expression was analyzed using qRT-PCR in ARPE-19 cells treated with control (siCtrl) or GGA2 (siGGA2) siRNA for 3 days. (c) ARPE-19 cells transfected with control (siCtrl) or GGA2 (siGGA2) siRNA were incubated with [^{35}S] methionine and cysteine for 120 min and were lysed with PBS containing 1% Triton X-100 and protease inhibitor cocktail followed by immunoprecipitation using anti-EGFR antibody. Approximately 0.5% of input was applied to the gel lane labeled with “lysate”. (d) Ratio of immunoprecipitated EGFR over total [^{35}S]-labeled proteins was quantified, then the value of siGGA2 was normalized to that of siCtrl. Data from three separate experiments are shown.

Supplementary Fig. S2

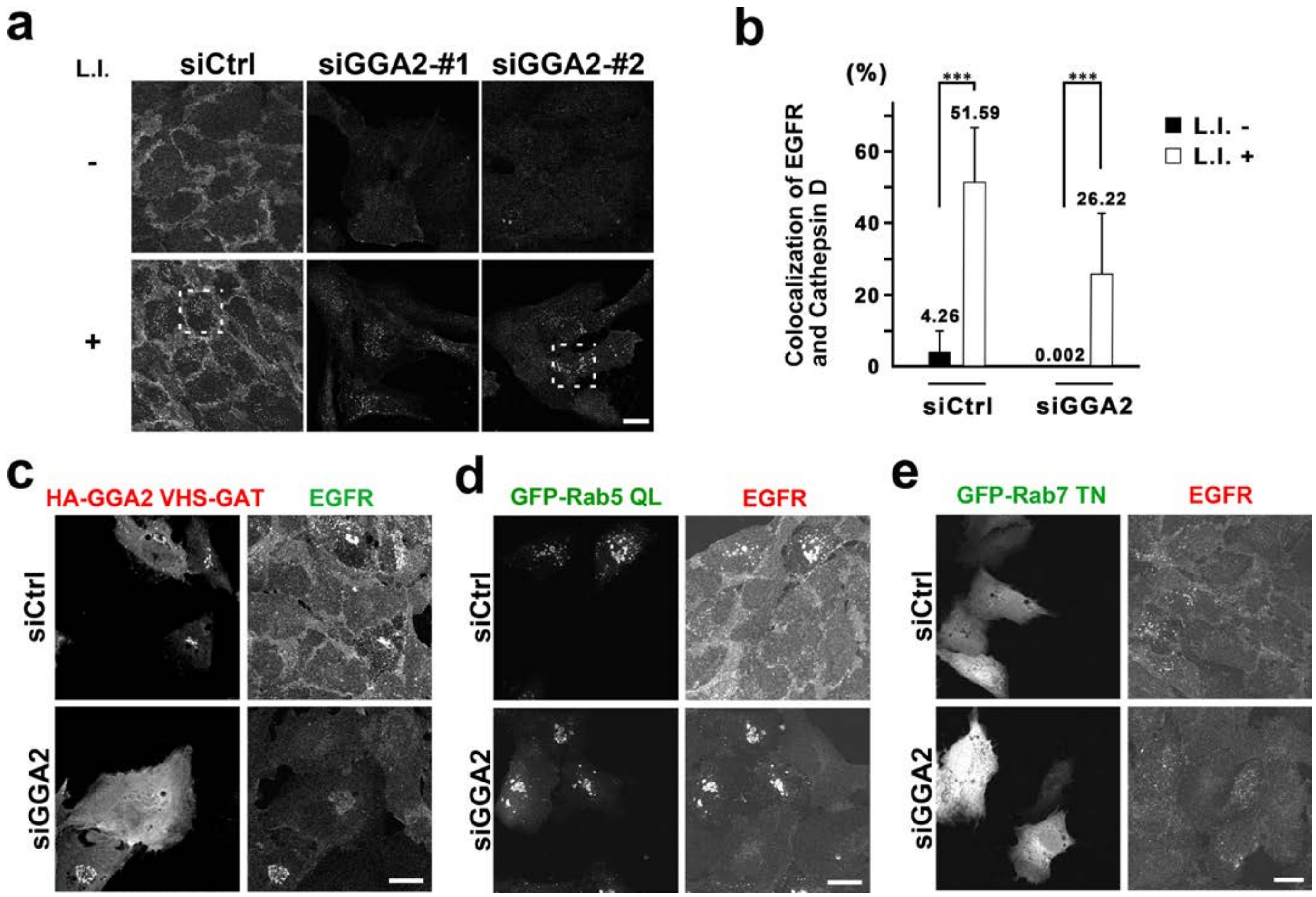

Supplementary Fig. S2. GGA2-depletion facilitates lysosomal degradation of EGFR via post-Golgi compartments

(a) Control (siCtrl) and GGA2-depleted (siGGA2#1 and siGGA2#2) ARPE-19 cells were treated with (+) or without (−) lysosomal protease inhibitors (L.I.) for 6 h, followed by fixation and double immunofluorescence staining with anti-EGFR and anti-cathepsin D antibodies. Boxed regions are magnified in the main Fig. 2c. Bar, 20 μm. (b) Percentages of double positive puncta for EGFR and cathepsin D over cathepsin D positive ones after incubation for 6 h with (+) or without (−) L.I. were measured in siCtrl and siGGA2 cells (mean ± SD; n = 15 cells for each group). Mean values for each group are indicated in the graph. ***: $p < 0.001$. (c-e) Control (siCtrl) or GGA2-depleted (siGGA2) ARPE-19 cells were transfected with expression vector containing the following mutants: HA-GGA2 VHS-GAT (siRNA resistant) (c), GFP-Rab5 QL (d), and GFP-Rab7 TN (e). Cells were fixed for immunofluorescence microscopy using anti-HA and/or anti-EGFR antibodies. Bars, 20 μm.

Supplementary Fig. S3

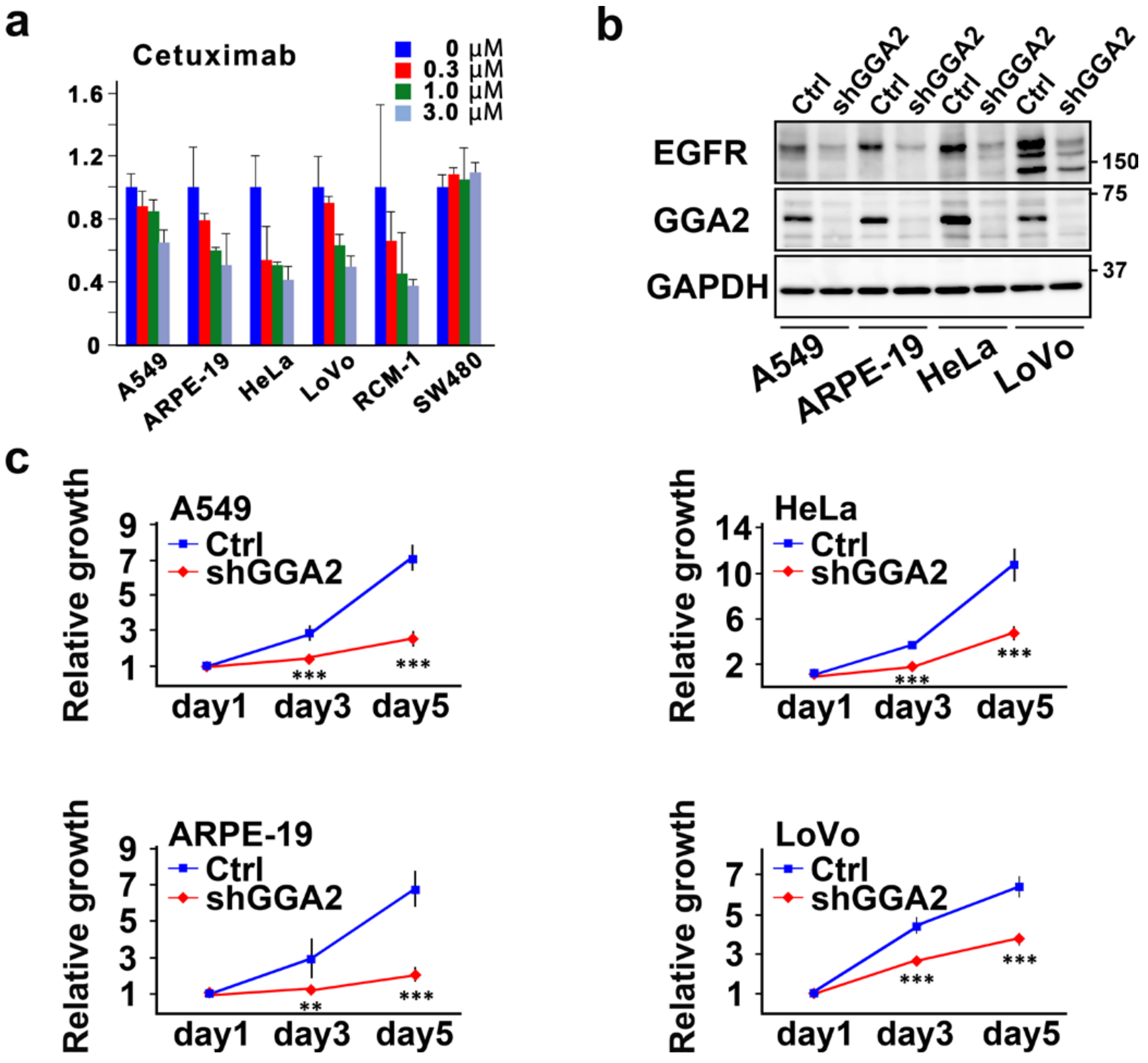

Supplementary Fig. S3. GGA2-depleted cell lines exhibit impaired cell growth (a) Six cell lines as indicated were cultured in the presence of the EGFR inhibitor cetuximab at indicated concentrations for 3 days, and surviving cells were quantified using Cell Counting Kit-8. SW480 cells were included as a control cell line with demonstrated EGFR-independent growth. (b) Stable GGA2-knockdown cells were generated using a lentiviral system in A549, ARPE-19, HeLa, and LoVo cells. Control (Ctrl) and GGA2-depleted (shGGA2) cells were lysed for western blot analyses with anti-EGFR, GGA2, and GAPDH antibodies. (c) Cell proliferation assays were performed in cultures of the generated cell lines. Data are presented as means ± SD of three experiments; $**P < 0.01$ and $***P < 0.001$.

Supplementary Fig. S4

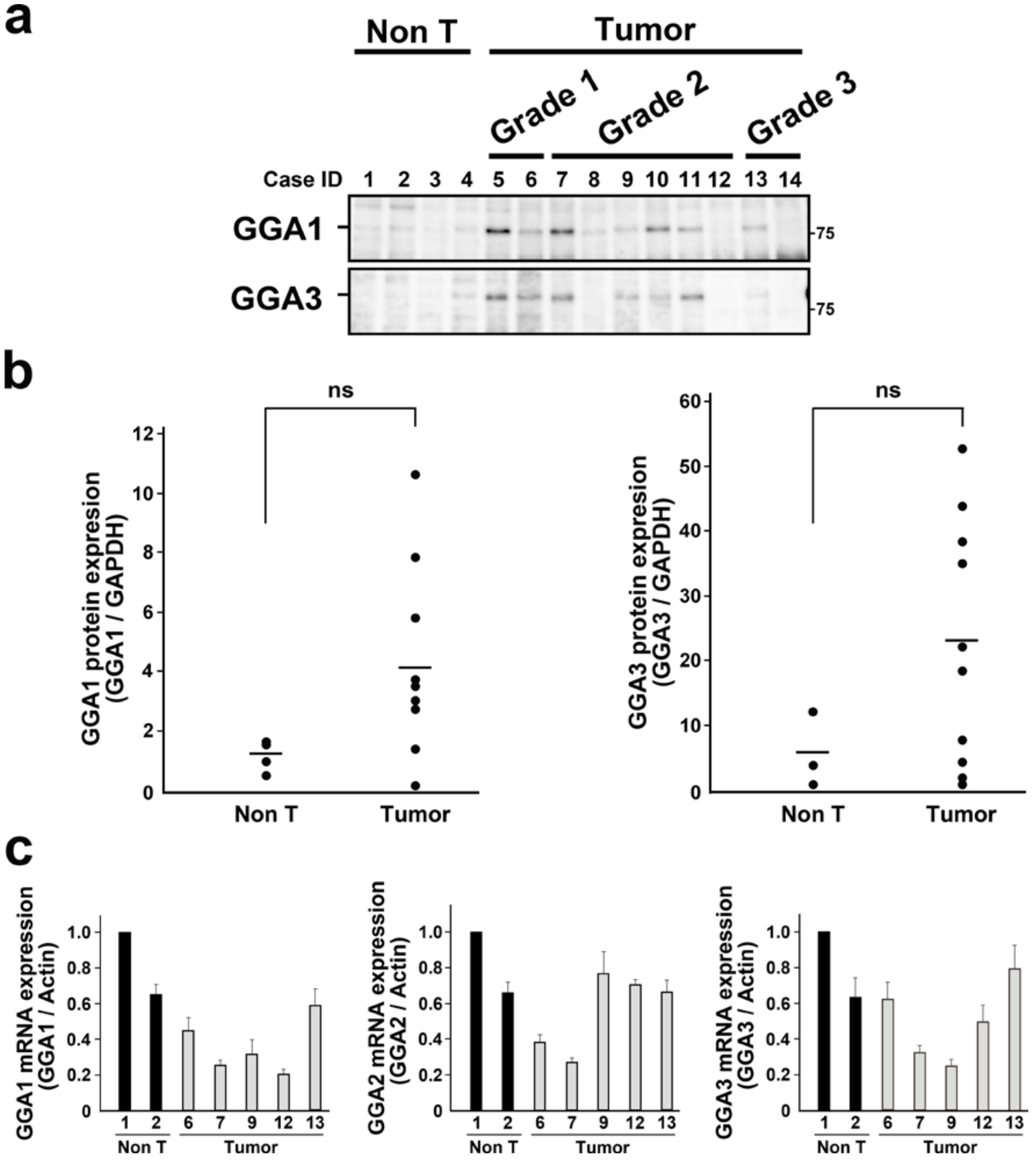

Supplementary Fig. S4. Protein and mRNA expressions of GGA1, GGA2, and GGA3 in HCC

(a) Western blot analysis of GGA1 and GGA3 in non-tumor (Non T) and tumor regions of HCCs. Histological grade is shown for each case. (b) Ratios of GGA1 or GGA3 to GAPDH were calculated and normalized to those of case #1. Statistical analysis was performed using Mann-Whitney *U*-test. ns: not significant. (c) Values of mRNA for GGA1, GGA2, and GGA3 were analyzed in selected patient cases using qRT-PCR. The case IDs are identical with those in the main Fig. 7a and S4a.

Supplementary Fig. S5

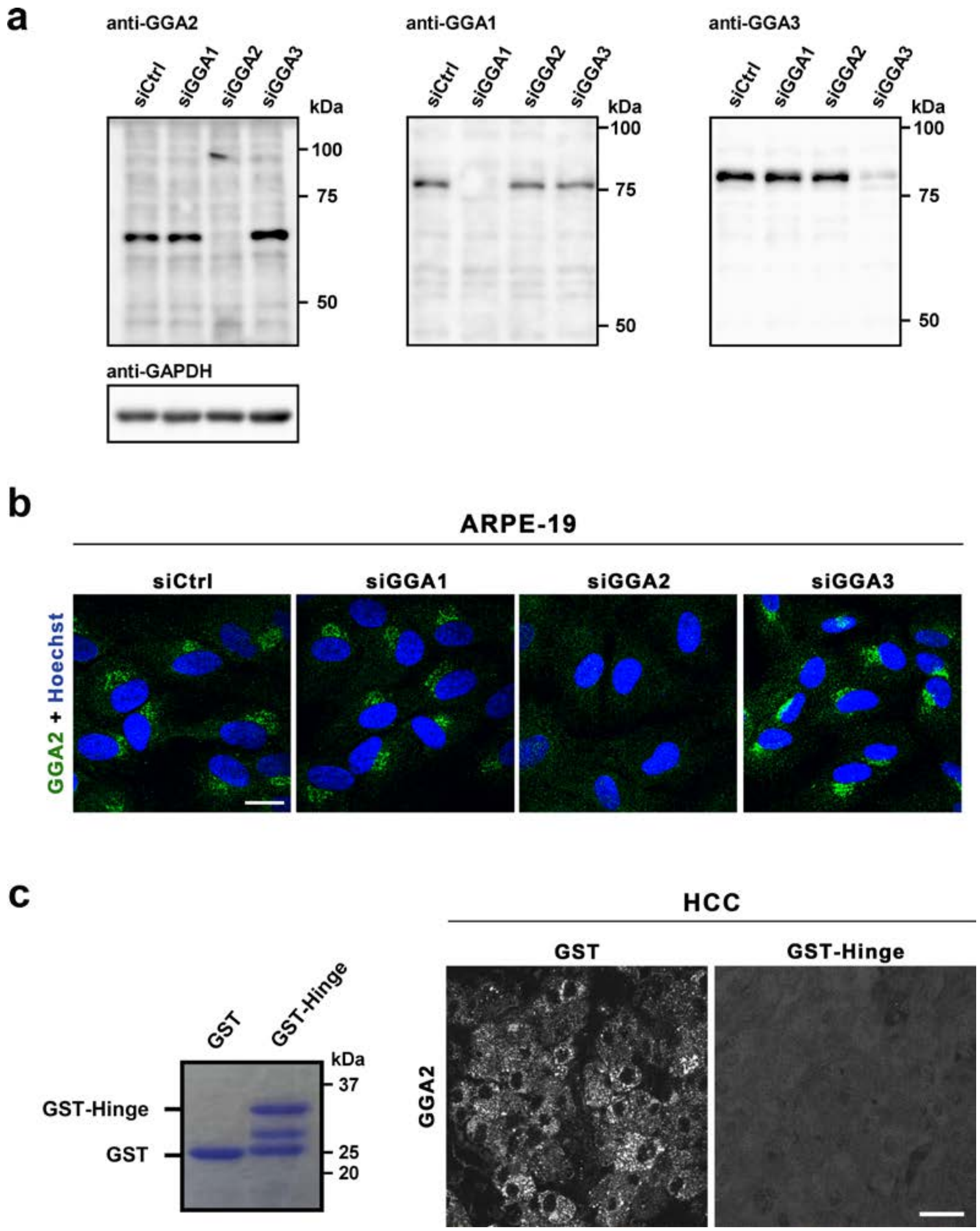

Supplementary Fig. S5. Characterization of the anti-GGA2 antibody used in immunohistofluorescence analyses of paraffin-embedded tissue arrays

(a and b) Control (siCtrl) or GGAs-depleted (siGGA1, siGGA2, and siGGA3) ARPE-19 cells were lysed for western blotting (a) or fixed for immunohistofluorescence analyses (b) using indicated antibodies. Nuclei were stained with Hoechst 33342 (blue). (c) Purified GST and GST fused with GGA2 hinge region (GST-Hinge) were analyzed in SDS-PAGE and stained with Coomassie Brilliant Blue (left). Paraffin sections of the HCC tissue array were immunostained using anti-GGA2 antibody preincubated with the GST or GST-Hinge. Result of a case with high expression levels of GGA2 is shown. Bars, 20 μm.

Supplementary Fig. S6

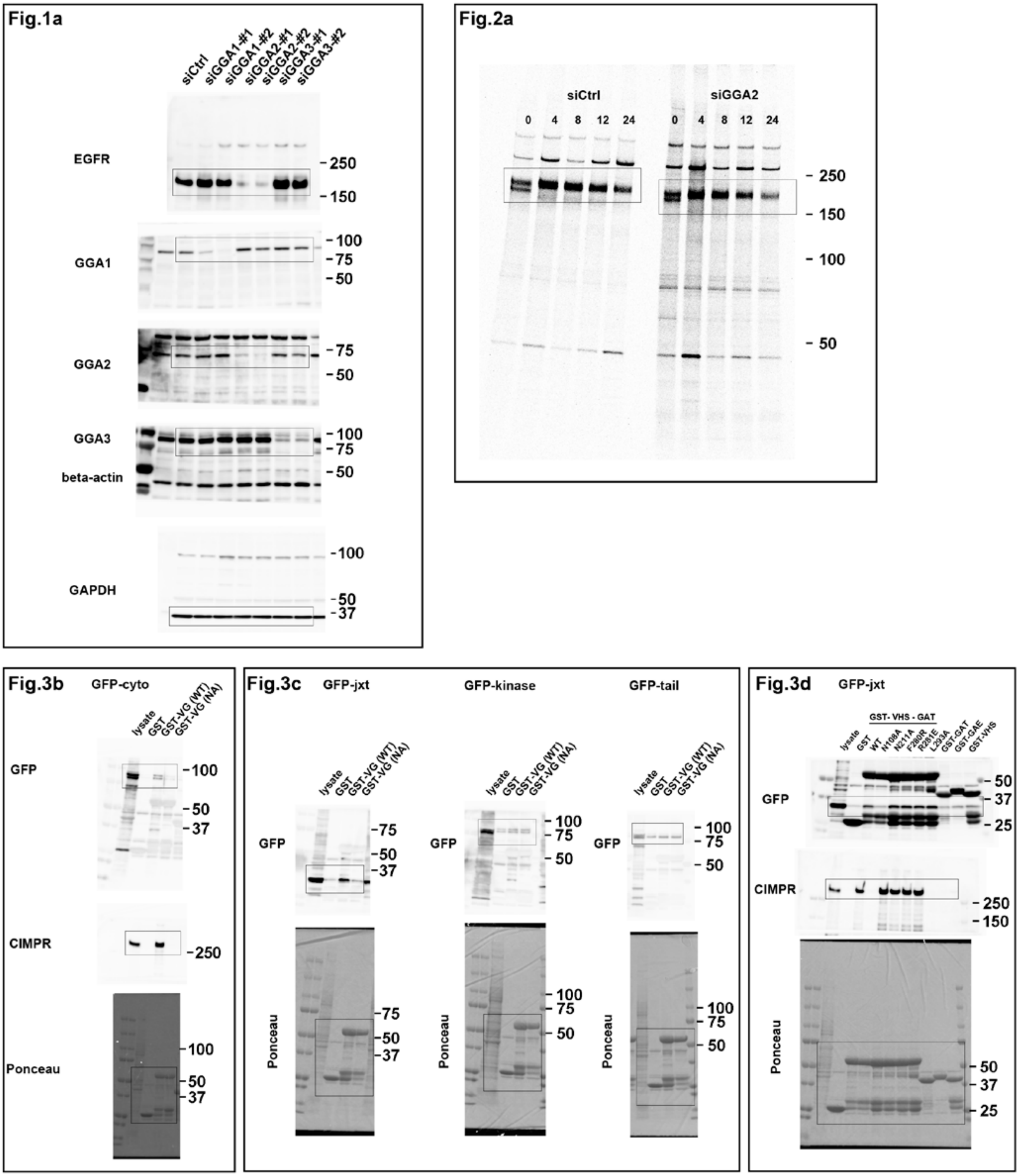

Supplementary Fig. S6. Uncropped gels/blots for main Figs. 1a, 2a, 3b, 3c, and 3d Boxed regions are used in the main figures. For some data, transferred membranes were horizontally cut for applying multiple antibodies. Lanes for unrelated samples are deleted.

Supplementary Fig. S7

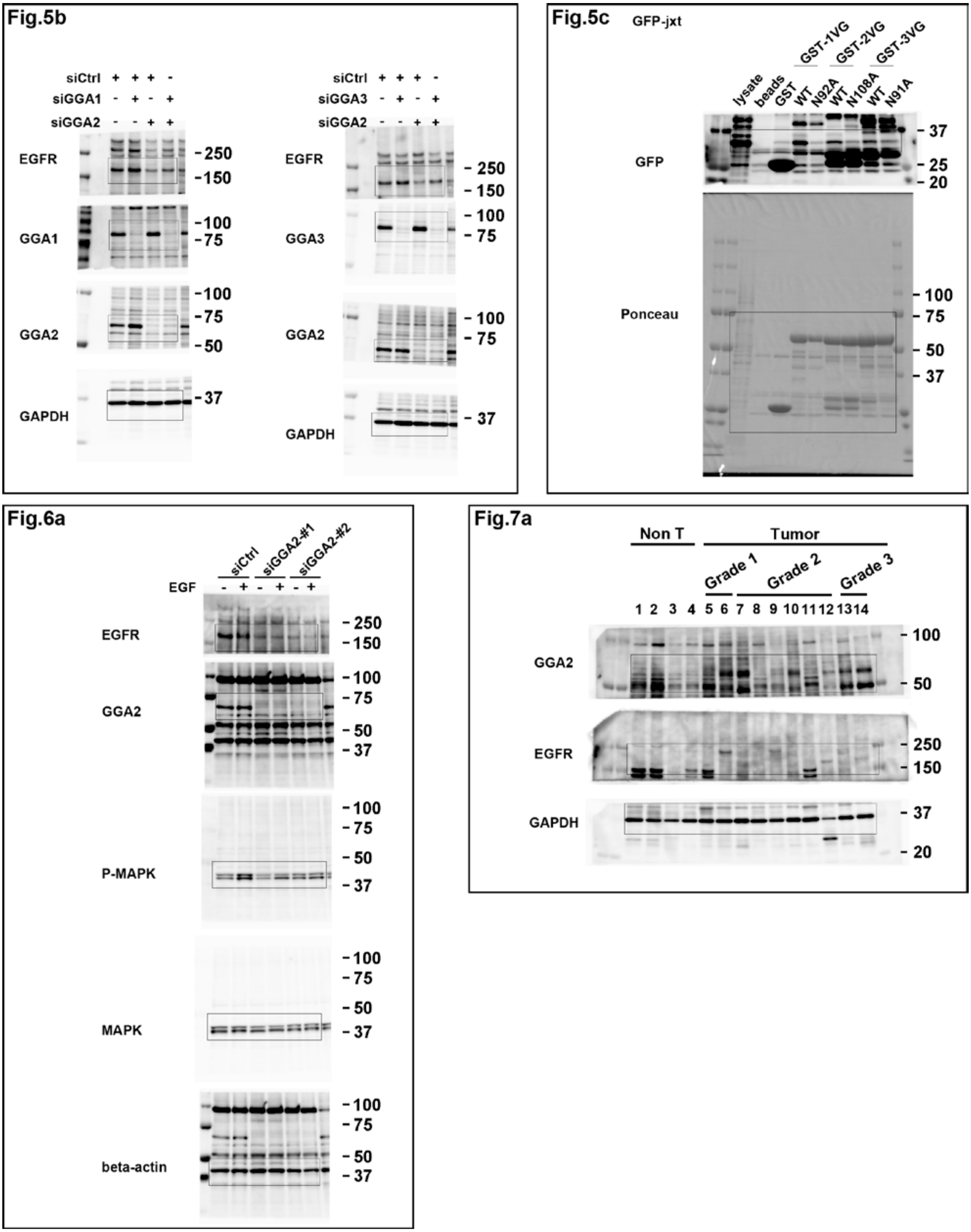

Supplementary Fig. S7. Uncropped gels/blots for main Figs. 5b, 5c, 6a, and 7a Boxed regions are used in the main figures. For some data, transferred membranes were horizontally cut for applying multiple antibodies. Lanes for unrelated samples are deleted.

Supplementary Table

Table S1. HCC sample information used for western blot analysis and qRT-PCR

Sample Number (case ID)	Protein ID	RNA ID	Diagnosis	Grade	Stage
#1 (CU0000000466)	CP601387	CR562345	n	na	na
#2 (CI0000005835)	CP508185	CR560916	n	na	na
#3 (CI0000008475)	CP565548		n	na	na
#4 (CI0000016506)	CP565687		n	na	na
#5 (sCU0000001197)	CP607175		HCC	G1 (wd)	IIIA
#6 (CI0000019939)	CP565722	CR562610	HCC	G1 (wd)	II
#7 (CU0000005407)	CP619427	CR561743	HCC	G2 (md)	II
#8 (CI0000019288)	CP548783		HCC	G2 (md)	I
#9 (CI0000020838)	CP565725	CR562534	HCC	G2 (md)	IV
#10 (CU0000005719)	CP622156		HCC	G2 (md)	IIIA
#11 (CI0000008358)	CP520741		HCC	G2 (md)	II
#12 (CU0000000996)	CP605491	CR562998	HCC	G2 (md)	IIIA
#13 (CI0000008275)	CP520428	CR561021	HCC	G3 (pd)	IIIA
#14 (CU0000012132)	CP641361		HCC	G3 (pd)	I

Modified from a data sheet from OriGene Technologies, Inc. Sample numbers correspond with those in Fig. 7 and Fig. S4. n, within normal limits; HCC, hepatocellular carcinoma; na, not applicable; wd, well differentiated; md, moderately differentiated; pd, poorly differentiated
